# Intraocular pressure screening during high-volume cataract surgery outreach in Ethiopia

**DOI:** 10.1186/s12886-022-02618-1

**Published:** 2022-10-05

**Authors:** Ian J. McClain, David M. Rooney, Geoffrey C. Tabin

**Affiliations:** 1 Larner College of Medicine, University of Vermont, 46 Colchester Avenue, Burlington, Vermont 05401 USA; 2 Himalayan Cataract Project, Waterbury, VT USA; 3 Department of Ophthalmology, Stanford University, Palo Alto, California USA

**Keywords:** Global ophthalmology, Glaucoma, Cataract, Blindness

## Abstract

**Introduction:**

Glaucoma is the leading cause of irreversible blindness worldwide and is often undetected in resource-limited settings. Early screening and treatment of elevated intraocular pressure (IOP) reduces both the development and progression of visual field defects. IOP screening in developing countries is limited by access to ophthalmic equipment, trained ophthalmic staff, and follow up. High-volume cataract surgery outreaches in resource-limited countries provide ample opportunity for glaucoma screening, intervention and follow up.

**Methods:**

This prospective cross-sectional study took place during a cataract outreach campaign sponsored by the Himalayan Cataract Project (HCP) in partnership with Felege Hiwot Hospital in Bahir Dar, Ethiopia, during April 5th – April 10th 2021. IOP was measured on the surgical eye of patients before undergoing small incision cataract surgery (SICS) using rebound tonometry with an iCare tonometer model IC100.

**Results:**

Intraocular pressure (IOP) was measured in 604 eyes of 595 patients who received SICS. Mean IOP was 12.1 mmHg (SD = 5.0 mmHg). A total of 29 patients had an IOP greater than 21 mmHg representing 4.8% of total IOP measurements. A total of 17 patients received oral acetazolamide prior to surgery to acutely lower IOP. Six of these patients had their surgery delayed due to elevated IOP and 9 patients received excisional goniotomy at the time of SICS. A temporal approach during SCIS was taken for all patients with elevated IOP to allow for possible trabeculectomy at a future date.

**Discussion:**

IOP screening during high-volume cataract outreach campaigns can be performed safely, accurately and on a large scale with minimal resources and supplemental training. Pre-operative IOP measurement can improve surgical care at the time of cataract surgery as well as help establish long-term follow up for patients with glaucoma.

## Introduction

Approximately 2.2 billion people worldwide have visual impairment; refractive error and cataracts are the most common cause of reversible blindness while glaucoma is the leading cause of irreversible blindness [1]. Early screening and treatment is particularly important for preventing the progression of glaucoma, which is generally asymptomatic until late in the disease course. The World Health Organization estimates that nearly 8 million cases of moderate or severe visual impairment or blindness from glaucoma could have been prevented or are untreated [2]. Epidemiological data also suggests undetected glaucoma is more common in resource poor settings in Africa and Asia compared to Europe [1]. Glaucoma screening in developing countries is limited by access to equipment, trained ophthalmic staff, and follow up.

Ethiopia has a high rate of blindness with a prevalence of approximately 1.6%. Almost 80% of these cases are thought to be treatable or preventable [3]. While data on the prevalence of glaucoma in Sub-Saharan Africa, including Ethiopia, is limited, it has been reported to be as high as 10.2% during community-screening events in Southwest Ethiopia [4]. A high rate of pseudoexfoliative glaucoma has also been described in Ethiopia, and pseudoexfoliation is anecdotally observed with considerable frequency by surgeons during cataract outreach events throughout the country [5].

The United States Preventative Services Task Force has determined that early screening and treatment of elevated intraocular pressure (IOP) reduces both the development and progression of visual field defects [6]. While mass screening programs have been performed on unselected populations in public areas, high-volume cataract surgery campaigns provide additional opportunity for IOP screening [4]. Cataract surgery campaigns are high-volume, safe and effective surgery efforts performed over a short period, typically a few days in a given location [7]. They have been implemented in several low and middle-income countries to increase cataract surgery rates in areas with limited access to eye care. The Himalayan Cataract Project (HCP) is a non-profit organization that supports professional eye care development and organizes cataract surgery outreach events throughout Africa and Asia. Since 2008, HCP has directly supported hundreds of cataract campaigns in Ethiopia resulting in over 70,000 surgeries to date.

In this study, we describe the distribution of pre-operative IOP measurements taken during an HCP cataract outreach event in Bahir Dar, Ethiopia in early April 2021. In addition to better characterizing the IOP and risk factors for glaucoma in this population of cataract patients, the goal of this study was to assess the feasibility of large-scale IOP screening during a cataract surgery outreach campaign.

## Methods

This prospective cross-sectional study received approval from the Institutional Review Board at Stanford University and was performed in accordance with the Declaration of Helsinki for human subjects research. Informed consent was obtained from patients or their legal guardian prior to participation in the study.

This study took place during a cataract outreach campaign sponsored by the Himalayan Cataract Project (HCP) in partnership with Felege Hiwot Hospital in Bahir Dar, Ethiopia, during April 5th – April 10th 2021. HCP is partnered with local providers and hospitals across Ethiopia and sponsors several high-volume cataract outreach campaigns per year. Thousands of cataract surgeries are provided to at-need patients through these campaigns.

Cataract patients were referred from the surrounding city and rural farming communities by ophthalmic technicians who routinely screen for cataracts. Patients interested in cataract surgery received free transportation to the hospital and overnight accommodation, provided by HCP. After arriving to the hospital, patients underwent additional examination by ophthalmic nurses using flashlights and portable slit lamps. For those patients with significant cataracts, visual acuity, intraocular pressure (IOP) and biometry measurements were then taken in anticipation of manual small incision cataract surgery (SICS) performed by HCP-affiliated ophthalmologists and partnering local ophthalmologists. All surgery was performed using SICS by a total of 5 ophthalmologists.

IOP was measured using rebound tonometry with an iCare tonometer model IC100. All measurements were performed by a medical student (IJM) who was trained to use the iCare tonometer by an HCP glaucoma specialist (DMR). Measurements were rechecked periodically by the attending glaucoma specialist and were found to be congruent with the student’s measurements.

## Results

Intraocular pressure (IOP) was measured in 604 eyes of 595 patients who subsequently received SICS. There were 330 males and 265 females. The mean age was 64 years and standard deviation (SD) was 13.2 years. Over 84% of eyes had either light perception or hand motion vision preoperatively (Table 1).

**Table 1:** Pre-operative visual acuity (VA) of the eyes for which intraocular pressure (IOP) was measured

*Pre-operative VA*	*# Patients*	*Percentage*
LP	174	28.8%
HM	338	56.0%
CF 1 m	59	9.8%
CF 2 m	10	1.6%
CF 3 m	4	0.7%
> CF 3 m or missing data	19	3.1%
**Total**	604	100%

**Fig. 1:**
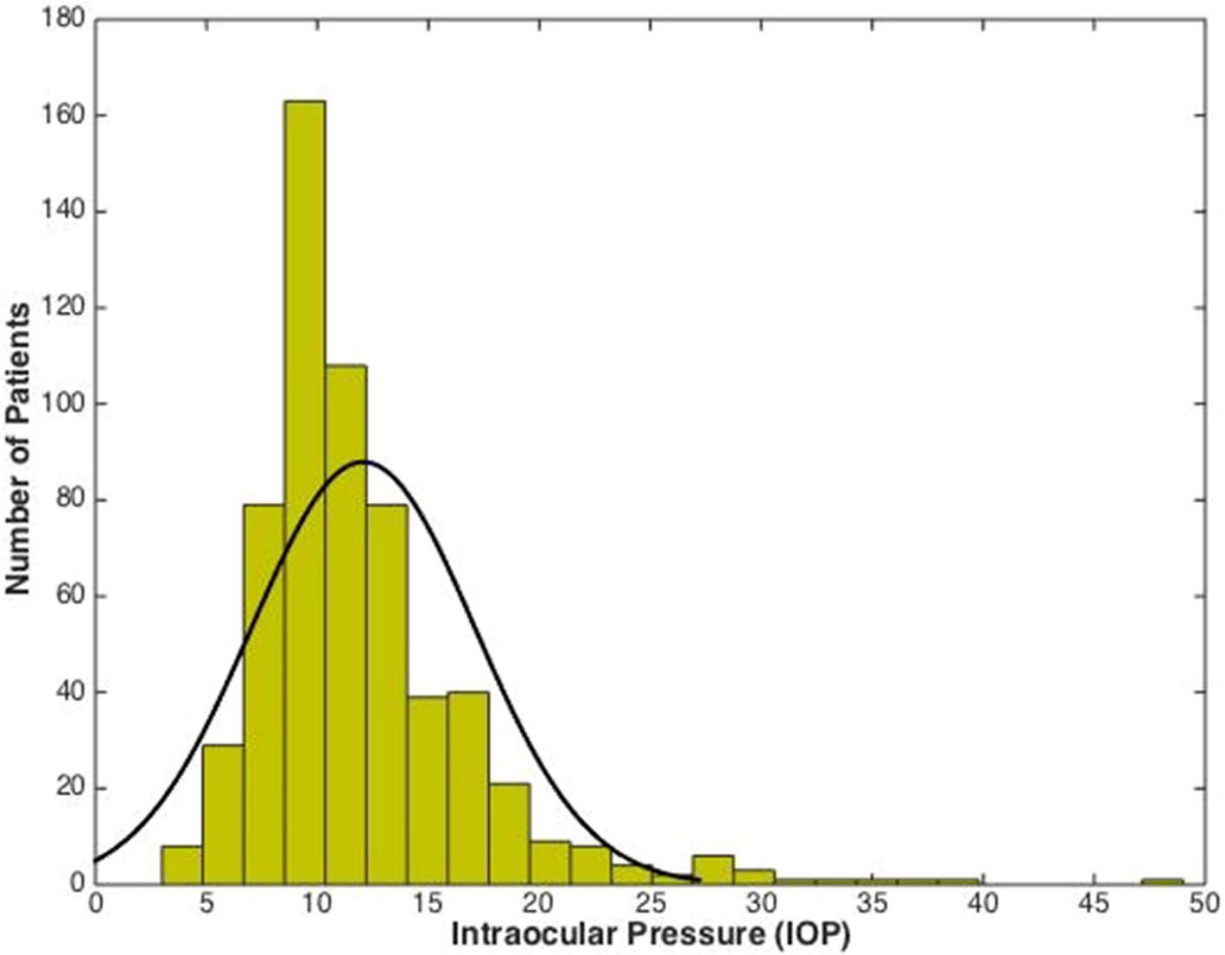
Histogram with distribution of intraocular pressure (IOP) plotted in 5 mmHg bins (gold bars) with overlaid normal density function (black line)

Mean IOP was 12.1 mmHg (SD = 5.0 mmHg). A total of 29 patients had an IOP greater than 21 mmHg representing 4.8% of total IOP measurements. The minimum IOP was 3 mmHg and maximum was 49 mmHg (see Fig. 1 for the complete distribution of IOP measurements).

A total of 17 patients received 500 mg of oral acetazolamide at least 1 hour prior to cataract surgery for an IOP equal or greater to 26 mmHg to reduce the risk of IOP-related complications such as suprachoroidal hemorrhage. Of these 17 patients, 6 patients had their surgery delayed until the following day for an IOP greater than 30 mmHg.

In addition to receiving SICS, a total of 9 patients also received minimally invasive glaucoma surgery (MIGS) with excisional goniotomy using a Kahook Dual Blade. There were no complications from goniotomy in these patients and no suprachoroidal hemorrhages were noted in patients with elevated IOP.

A temporal approach was taken for all SICS patients with elevated IOP > 21 mmHg in case trabeculectomy was warranted at a future date. All patients with elevated IOP were also scheduled for 1 month-follow up for clinical examination and completion of glaucoma workup, including fundus examination, gonioscopy, and IOP monitoring.

## Discussion

Elevated IOP is the major and only known modifiable risk factor for glaucoma [8]. Mean IOP varies based on geographic location, ethnicity and the tonometer used [9]. Research examining the distribution of IOP and prevalence of glaucoma in Ethiopia is limited; however, a sample of 459 individuals screened in public shopping centers, 10.2% were diagnosed with glaucoma after screening pressure, visual fields and fundus exam data were collected [4]. Larger, population studies in sub-Saharan Africa have suggested an overall prevalence of glaucoma ranging from 4.5 to 5.3% [6].

In our study’s population of cataract surgery patients, mean preoperative IOP was 12.1 mmHg with 29 eyes (4.8%) having an IOP above 21 mmHg. As is typical of cataract surgery outreaches, pre-operative visual field testing and fundoscopic examinations were not possible because of the severe density of cataracts (pre-operative visual acuity was count fingers at 1m  or worse in over 94% of patients). Thus, we were unable to diagnose glaucoma prior to surgery. However, IOP screening was feasible and influenced patient care. A total of 17 patients received oral acetazolamide prior to surgery to acutely lower IOP, 6 patients had their surgery delayed to the following day for IOP-related safety concerns, and 9 patients had additional surgery with excisional goniotomy using a MIGS approach. All patients were treated as glaucoma suspects and scheduled for follow up to undergo complete ophthalmic examination and future monitoring of IOP. In addition, a temporal approach was taken for all SICS patients with elevated IOP in case trabeculectomy was warranted at a future date.

This study had limitations, which warrant mentioning. Rebound tonometry using an iCare model IC100 was employed to measure IOP. While IOP measured by the iCare tonometer generally correlate well with Goldmann applanation tonometry, studies comparing the two have been mixed regarding the devices predilection to either slightly underestimated or overestimate IOP [10, 11]. In 2017, Gao et al. found the iCare tends to correlate well with IOP measurements less than 23 mmHg, although it may underestimate the pressure above 23 mmHg [10]. Badakere et al. recently compared the newest iCare model, the IC200, to Goldmann applanation tonometry in 193 eyes and found that the device slightly overestimates IOP, with the overestimation increasing as baseline IOP increases [11]. We were unable to use gold standard Goldmann applanation to measure IOP in this study; however, we find the iCare tonometer to be a excellent screening tool even though it may be less useful for monitoring IOP in long-term glaucoma treatment.

Goldmann applanation is often not available in settings where cataract outreach campaigns are performed. In contrast, iCare devices are portable, require limited training, no anesthetic eye drops and measurements can be collected in a shorter period of time. The device can be sanitized quickly and is particularly well suited for the high-volume and fast paced nature of cataract surgery campaigns.

Although elevated IOP is the most significant risk factor for glaucoma, eyes with normal IOP may still develop glaucoma [12]. While IOP screening is an excellent first step to screen patients for possible glaucoma, a complete ophthalmic examination including visual fields and fundus examination on all patients following cataract surgery is necessary to diagnose the disease.

Finally, this study was limited in not assessing post-operative IOP. It is possible a large percentage of elevated IOP was due to phacomorphic glaucoma from hypermature cataracts. It will be important to assess post-operative IOP in future studies screening for glaucoma in high-volume cataract campaigns.

## Conclusions

IOP screening can be performed safely, accurately and on a large scale with minimal resources and training. Furthermore, IOP measurements can make a tangible difference on surgical care and patient follow up in cataract outreach events. IOP screening during cataract surgery campaigns provide additional opportunity for glaucoma screening, intervention and follow up.

## Data Availability

The datasets generated and/or analyzed during the current study are not publicly available due but are available from the corresponding author on reasonable request.
